# Clinical Outcomes of Immediate and Delayed Composite Restorations After Pulp Capping with Biodentine: A Systematic Literature Review

**DOI:** 10.3390/jfb17050241

**Published:** 2026-05-10

**Authors:** Margarita Aleksiuk, Ana Kostenkova, Saulius Drukteinis

**Affiliations:** Institute of Dentistry, Faculty of Medicine, Vilnius University, Žalgirio Str. 115, LT-08217 Vilnius, Lithuania; margarita.aleksiuk@mf.stud.vu.lt (M.A.); ana.kostenkova@mf.stud.vu.lt (A.K.)

**Keywords:** Biodentine, vital pulp therapy, direct pulp capping, indirect pulp capping, composite restoration, temporary restoration, deep carious lesions

## Abstract

Background: Biodentine is widely used in vital pulp therapy due to its bioactivity and biocompatibility. However, treatment success depends not only on the material but also on the restorative approach. Clinically, Biodentine may be used as a temporary bulk restoration before delayed placement of a composite or immediately covered with a definitive composite. Aim: To evaluate clinical outcomes reported for delayed composite placement after temporary Biodentine restoration and immediate composite restoration following Biodentine pulp capping in permanent teeth. Methods: A systematic review was conducted in accordance with PRISMA guidelines and registered with PROSPERO (CRD420261325248). Searches were performed in multiple databases. Clinical studies on Biodentine pulp capping reporting outcomes for either delayed or immediate composite restoration were included. Study selection, data extraction, and quality assessment were performed by two reviewers using Joanna Briggs Institute tools. Fourteen studies (8 randomized controlled trials and 6 cohort studies) were included. Results: Considerable heterogeneity was observed in study design and clinical protocols. Most included studies evaluated one of the two strategies separately, so the review results could not be interpreted as a direct comparison. In direct pulp capping, success rates ranged from 74–100% (delayed) and 79–100% (immediate). In indirect pulp capping, success rates ranged from 77.8–88% (delayed) and 80–95.2% (immediate). Meta-analysis was not feasible. Conclusion: Based on low certainty of evidence, both strategies show favorable outcomes, but current evidence does not support the superiority of either approach. Further well-designed comparative studies are needed.

## 1. Introduction

Vital pulp therapy (VPT) is a minimally invasive approach aimed at preserving pulp vitality and includes procedures such as direct and indirect pulp capping. Biodentine, a calcium silicate-based material, is widely used for this purpose due to its favorable biocompatibility, bioactivity, and relatively short setting time [1]. However, the success of pulp-capping procedures depends not only on the properties of the capping material but also on the subsequent coronal restoration. An adequate coronal seal is essential to prevent microleakage and bacterial contamination, which are key factors associated with treatment failure [2,3,4]. At the same time, clinical outcomes of pulp capping are influenced by multiple additional factors, including pulp diagnosis, hemostasis control, and characteristics of the lesion, although their relative importance remains uncertain and inconsistently reported in the literature.

The optimal restoration strategy following pulp capping remains a topic of ongoing debate. Using Biodentine as a temporary bulk restoration has been suggested as a possible approach that may allow further maturation of the material before placement of the final restoration [5]. However, this approach may present certain limitations. Biodentine may exhibit lower mechanical strength and wear resistance compared to composite materials [6]. In addition, delayed restoration protocols may introduce practical limitations, including the need for multiple visits, potential patient noncompliance, and risk of pulp re-exposure during material reduction. Immediate composite restorations over Biodentine provide a definitive coronal seal in a single visit but may expose the pulp to thermal stress during light curing and polymerization shrinkage, particularly in cases where the remaining dentin thickness is minimal [7].

Although clinical studies have reported favorable outcomes for both immediate and delayed restorative approaches, the available evidence is heterogeneous, and most studies evaluate only one strategy rather than directly comparing both within the same study design. As a result, it remains unclear whether one restorative approach offers superior clinical outcomes over the other. The aim of this systematic review was to evaluate the available clinical evidence on restorative strategies following Biodentine pulp capping in permanent teeth with deep carious lesions, specifically comparing delayed definitive composite restoration after temporary Biodentine bulk restoration with immediate definitive composite restoration.

## 2. Materials and Methods

### 2.1. Protocol and Registration

This systematic review was conducted and reported in accordance with the Preferred Reporting Items for Systematic Reviews and Meta-Analyses (PRISMA) statement [8] and the PRISMA checklist was used to ensure complete reporting of the review process (File S1). The review protocol was registered in the International Prospective Register of Systematic Reviews (PROSPERO) under the registration number CRD420261325248.

### 2.2. Focus Question

The review question was formulated according to the PICO framework as follows: In mature or immature permanent teeth with deep carious lesions treated with Biodentine for direct or indirect pulp capping (P), does delayed definitive composite restoration after temporary Biodentine bulk restoration (I), compared with immediate definitive composite restoration (C), result in different clinical and/or radiographic outcomes (O) [9]?

### 2.3. Eligibility Criteria and Search Strategy

The eligibility criteria were defined according to the PICOS framework as follows: mature or immature permanent teeth with deep carious lesions treated with Biodentine for indirect and/or direct pulp capping (P); delayed definitive composite restoration after temporary Biodentine bulk restoration (I); immediate definitive composite restoration after Biodentine pulp capping (C); clinical and/or radiographic outcomes (O); and clinical studies, including randomized controlled trials and observational studies (S).

Clinical studies were included if they evaluated Biodentine used for indirect and/or direct pulp capping in mature or immature permanent teeth with deep carious lesions and clearly reported one of the following restorative strategies: delayed definitive composite restoration after temporary Biodentine bulk restoration or immediate definitive composite restoration after Biodentine pulp capping. Eligible studies were required to report clinical and/or radiographic outcomes and to include a minimum follow-up period of 6 months. Studies were excluded if they involved traumatic or other non-carious pulp exposures, primary teeth, animal or in vitro models, case reports, narrative reviews, systematic reviews, or meta-analyses, or if the restorative protocol or Biodentine-specific outcomes could not be clearly identified.

A comprehensive electronic literature search was conducted in PubMed/MEDLINE, Web of Science, Scopus, ScienceDirect, the Cochrane Library, and Google Scholar between 1 October and 30 November 2025. No restrictions were applied regarding year of publication; however, only studies published in English were considered. The search strategy combined terms related to pulp capping and vital pulp therapy, Biodentine and calcium silicate-based materials, restorative strategy, and treatment outcomes. The full search strategy for each database is presented in Table 1.

Two reviewers independently screened the titles and abstracts of the retrieved records. After duplicate removal using Mendeley reference management software version 2.144.0 (Elsevier, Amsterdam, The Netherlands), potentially eligible articles were assessed in full text according to the predefined inclusion and exclusion criteria outlined in Table 2. Any disagreements regarding study selection were resolved through discussion with a third reviewer. Inter-reviewer agreement during the study selection process was assessed using Cohen’s kappa coefficient, which indicated substantial agreement (κ = 0.61).

### 2.4. Risk of Bias and Certainty of Evidence Assessment

The methodological quality of the included studies was assessed using the Joanna Briggs Institute (JBI) critical appraisal tools for randomized controlled trials and cohort studies, as appropriate for each study design. Each study was evaluated across the relevant appraisal domains, with responses recorded as “Yes”, “No”, “Unclear”, or “Not Applicable”. Overall risk-of-bias judgments were made by considering the pattern of responses across domains. Particular weight was given to domains related to randomization, allocation concealment, baseline comparability, outcome assessment, follow-up completeness, and control of confounding. Studies were classified as low risk when most domains were rated positively, and no critical domain raised major concern; moderate risk when some important domains were rated as unclear or negative but without major threat to validity; and high risk when several critical domains were rated as negative or unclear. Two reviewers independently performed the assessments, and any disagreements were resolved through discussion with a third reviewer.

The certainty of the evidence was assessed using the GRADE approach [10], taking into account risk of bias, inconsistency across study protocols and outcome definitions, indirectness of comparisons, imprecision due to small sample sizes, and the inability to perform a meta-analysis.

### 2.5. Data Extraction

The authors extracted data and collected the following principal characteristics from each included study: authorship, year, country of publication, tooth type, sample size, details of the pulp-capping procedure, type of restoration, and follow-up duration.

## 3. Results

### 3.1. Selected Studies

**Figure 1:**
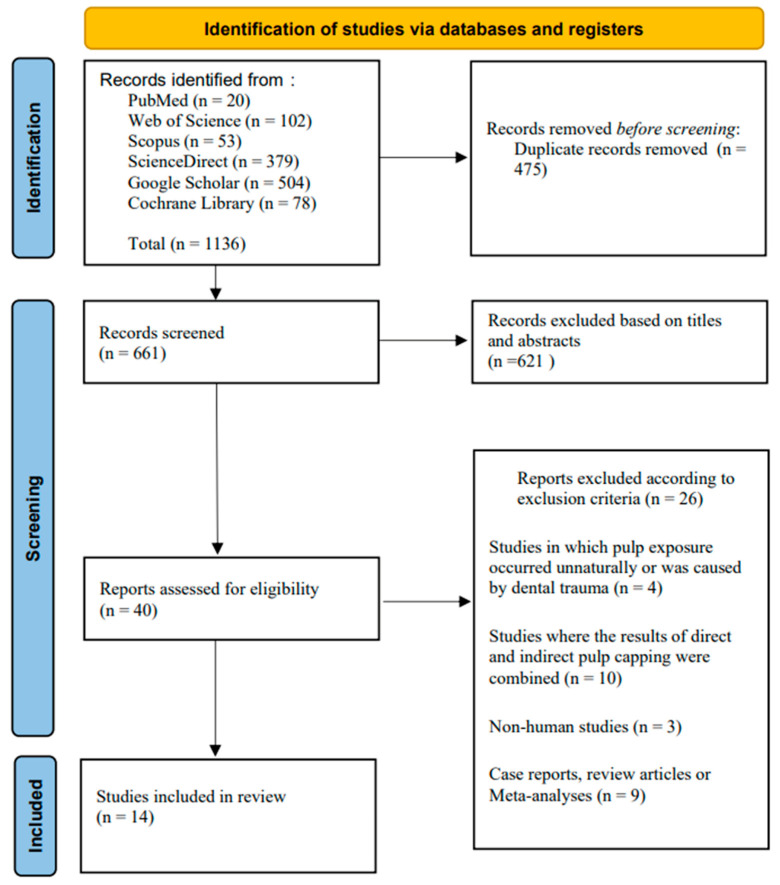
PRISMA flowchart outlining search strategy.

The study selection process is presented in the PRISMA flowchart (Figure 1). Following the electronic search and removal of duplicates, 40 studies were screened for eligibility. After title and abstract screening, 26 records were excluded based on the predefined exclusion criteria. Ultimately, 14 studies were included in the review [1,11,12,13,14,15,16,17,18,19,20,21,22,23].

### 3.2. Study Characteristics

The general characteristics of the included studies are summarized in Table 3. All studies evaluated clinical and/or radiographic outcomes of indirect pulp capping (IPC) or direct pulp capping (DPC) using Biodentine, followed by either Biodentine bulk restorations or immediate composite restorations. Considerable variability was observed among the included studies. The mean age of patients ranged from 8.1 to 42.02 years. Both immature and mature permanent teeth were included. The main inclusion criteria were vital pulp or reversible pulpitis, a positive pulp test response, a deep carious lesion penetrating more than three-quarters of the way into the dentine, and no PA changes on the radiograph. Some studies included only systemically healthy patients and excluded individuals taking corticosteroids or statins, as well as pregnant women [11,18,20,21,22]. While most studies excluded teeth with PA changes, Parinyaprom et al. excluded teeth with prominent radiolucency in the furcation or periapical region, internal or pathologic external root resorption, or pulp calcification, but allowed early periapical lesions such as widened PDL space or condensing osteitis [19]. Seven studies [1,11,12,16,19,21,22] included in the investigation all groups of teeth, two of which [19,21] investigated permanent teeth with open and closed apices, while seven studies [13,14,15,17,18,20,23] focused solely on posterior teeth, three studies focused only on mature molars [13,17,18] and two studies focused on immature permanent molars [14,23]. All treated teeth had deep carious lesions approaching the pulp and were considered restorable. While most studies focused on initial caries treatment, four included teeth with secondary caries as well [1,16,17,21]. All procedures were performed with local anesthesia under rubber-dam isolation.

### 3.3. Risk of Bias and Certainty of Evidence

Fourteen studies were included and assessed using the JBI critical appraisal tools (Table 4 and Table 5): eight randomized controlled trials [11,12,14,18,19,20,22,23] and six cohort studies [1,13,15,16,17,21]. The risk of bias was assessed as moderate in nine studies [1,13,15,16,17,20,21,22,23], low in three studies [11,12,19], and high in two studies [14,18]. The main limitations included a lack of blinding, unclear allocation concealment, incomplete follow-up, and potential bias in outcome assessment.

The certainty of evidence for the comparison between immediate composite restoration and delayed restoration using Biodentine as a bulk temporary material after Biodentine pulp capping was judged to be low (Table 6). Although the review included randomized controlled trials and cohort studies, the risk of bias was a concern because only three studies were assessed as low risk, nine as moderate, and two as high risk. Methodological inconsistencies were present due to patient age, tooth type and maturity, caries removal technique, timing of final restoration, and follow-up duration. One of the main issues is indirectness, as most studies did not directly compare the two restorative strategies within the same study population, and therefore, no meta-analysis could be performed.

### 3.4. DPC—Delayed Biodentine Approach

Across the included studies using the delayed Biodentine bulk approach for direct pulp capping (DPC), similar inclusion criteria were applied to select teeth with favorable pulpal conditions [1,13,14,17,21,22]. All studies included permanent teeth with deep carious lesions resulting in pulp exposure during caries excavation, with a clinical diagnosis of normal pulp or reversible pulpitis and positive responses to pulp sensibility tests. Teeth presenting with spontaneous pain, swelling, fistula, abnormal mobility, or tenderness to percussion were generally excluded. However, the characteristics of pulp exposures varied between studies. Two studies included only small exposures, typically <2 mm in diameter [1,14], whereas other studies accepted a wider range of exposure sizes.

Bleeding control criteria also differed. Two studies required that pulpal bleeding be controlled within a specific time frame, typically between 3 and 10 min [13,22], whereas four studies did not specify the time required to achieve hemostasis [1,14,17,21]. In all studies, procedures were performed under local anesthesia and rubber dam isolation.

Considerable heterogeneity was observed in patient age, tooth maturity, and tooth type. Three studies [1,21,22] included both anterior and posterior teeth, and Lipski et al. [21] additionally investigated immature and mature permanent teeth. Three studies [13,14,17] focused exclusively on molars, including one study by Katge et al. that specifically evaluated immature permanent molars [14].

All studies followed a delayed restorative protocol. Biodentine was initially used as a bulk temporary restoration and subsequently reduced to serve as a base before placement of the final composite restoration. The interval between initial placement and definitive restoration ranged from 1 week [22] to 3 months [14].

Reported success rates for DPC using the delayed Biodentine approach were generally high (Table 7), ranging from 74% to 100% [1,14]. High success rates were reported in studies involving younger patients and in those including smaller pulp exposures, such as the study by Katge et al., which reported a 100% success rate at 12 months [14]. Lower values were observed in studies with older patient populations or longer follow-up periods, for example, Drouri et al. reported a success rate of 74% at 6 months [1]. The only study by Lipski et al. evaluated both restorative strategies within the same population and reported success rates of 78.4% for the delayed Biodentine approach [21].

### 3.5. DPC—Immediate Composite Approach

Most studies evaluating direct pulp capping (DPC) followed by immediate composite restoration included permanent teeth with deep carious lesions that exposed the pulp during caries excavation, with a clinical diagnosis of normal pulp or reversible pulpitis and positive responses to pulp sensibility tests. Teeth presenting with spontaneous pain, swelling, fistula, abnormal mobility, or tenderness to percussion were excluded. However, the characteristics of pulp exposures varied between studies. Two trials included only small exposures, typically ≤2–2.5 mm in diameter [19,23], whereas other studies accepted a wider range of exposure sizes [15,16,18,20,21].

Bleeding control criteria also differed across studies. Many studies required that pulpal bleeding be controlled within a defined time period, typically between 1 and 2 min and 10 min. Six studies reported clearly defined hemostasis criteria [15,16,18,19,20,23], whereas Lipski et al. did not specify the time required to achieve bleeding control [21]. In all studies, procedures were performed under local anesthesia and rubber dam isolation.

Considerable heterogeneity was observed in patient age, tooth maturity, and tooth type. Three studies [16,19,21] included both anterior and posterior teeth, while Lipski et al. and Parinyaprom et al. additionally investigated immature and mature permanent teeth. Four studies [15,18,20,23] focused exclusively on posterior teeth; among these, Brizuela et al. [23] included both mature and immature permanent molars.

In all studies, definitive restorations were performed immediately after Biodentine placement, most commonly with adhesive composite restorations [15,16,18,19,20,21,23]. Reported success rates for immediate composite restoration following DPC ranged from 79% to 100% (Table 8) [18,23]. High success rates were reported in studies involving younger patients and in those including smaller pulp exposures, such as the study by Brizuela et al., which reported a 100% success rate at 12 months [23]. Lower values were observed in studies with older patient populations or longer follow-up periods, for example, Peskersoy et al. reported approximately 79% success after 3 years [18] The only study that evaluated both restorative strategies within the same study population reported success rates of 85.7% for immediate composite restoration after 1–1.5 years [21].

### 3.6. IPC—Delayed Biodentine Approach

Indirect pulp capping (IPC) procedures were performed exclusively on mature permanent teeth, predominantly in adult patients. All included studies evaluated teeth with deep carious lesions approaching the pulp, in which a layer of caries-affected dentine was intentionally preserved to protect the pulp tissue.

Inclusion criteria were generally similar across studies. Hashem et al. [11,12] included teeth with deep carious lesions extending into the inner third of dentine, clinical signs of reversible pulpitis, positive pulp sensibility tests, and absence of periapical radiographic changes. Drouri et al. [1] included teeth with deep or very deep carious lesions without signs of irreversible pulpitis. These criteria suggest that IPC was primarily performed in teeth with favorable pulpal conditions.

Caries removal techniques varied slightly across studies but generally adhered to minimally invasive principles. Selective removal of infected dentine was performed using rotary instruments, and in Hashem et al.’s studies, chemo-mechanical caries removal (e.g., Carisolv) was used to preserve affected dentine near the pulp [1,11,12]. Despite differences in instrumentation, the underlying approach was consistent across studies.

Differences were observed in the timing of definitive restoration. In the studies by Hashem et al., cavities were initially restored with Biodentine, followed by placement of the final composite restoration after one month using a closed-sandwich technique [11,12]. A shorter delay was reported by Drouri et al., where Biodentine was used as a bulk temporary restoration, and the final composite restoration was placed after two weeks [1].

Reported success rates for IPC using the delayed Biodentine approach ranged from 77.8% to 88% [1,11] (Table 9). These findings should be interpreted with caution due to heterogeneity in study populations, clinical protocols, and outcome assessment criteria.

### 3.7. IPC—Immediate Composite Approach

Indirect pulp capping (IPC) with immediate composite restoration was performed exclusively on mature permanent teeth, predominantly in adult patients. All included studies evaluated teeth with deep carious lesions approaching the pulp, in which a layer of caries-affected dentine was intentionally preserved to protect the pulp tissue.

Yavuz et al. [20] included systemically healthy patients with deep dentinal caries, positive pulp sensibility tests, and absence of clinical signs such as spontaneous pain, swelling, or fistula. Similarly, Kusumvalli et al. [15] included teeth with positive sensibility tests and no spontaneous or lingering pain, no tenderness to percussion, and no sinus tract. These criteria suggest that IPC was primarily performed in teeth with favorable pulpal conditions.

Caries removal techniques varied slightly but generally followed minimally invasive principles. Yavuz et al. [20] used a combination of high-speed diamond burs and low-speed carbide burs, while Kusumvalli et al. applied a stepwise excavation technique using rotary instruments and a spoon excavator. Despite differences in instrumentation, the underlying approach remained consistent: to remove infected dentine while preserving unaffected dentine near the pulp.

In both studies, definitive restoration was placed immediately after IPC using adhesive composite materials [15,20]. Reported success rates were high (Table 10), with Yavuz et al. reporting 95.2% success at 6 and 12 months [20], and Kusumvalli et al. reporting 80% success at 12 months [15]. However, interpretation of the latter result is limited due to the small sample size (n = 5).

## 4. Discussion

This systematic review evaluated the available clinical evidence on immediate definitive composite restoration versus delayed composite placement after temporary Biodentine bulk restoration following pulp capping in permanent teeth. Both restorative approaches were associated with generally favorable clinical outcomes. However, interpretation of these findings is limited by substantial clinical and methodological heterogeneity and by the predominance of indirect comparisons across study populations.

Most included studies limited treatment to teeth with normal pulp or reversible pulpitis, which are generally considered more favorable conditions for vital pulp therapy because of lower levels of pulpal inflammation [24]. However, the relationship between the clinical diagnosis and the actual histological condition of the pulp remains uncertain. Previous studies have shown that clinical symptoms do not always reflect the true extent of pulpal inflammation, and teeth diagnosed with irreversible pulpitis may still contain histologically healthy pulp tissue [25]. This suggests that some degree of baseline misclassification may have influenced the reported outcomes. At the same time, symptom severity may still have prognostic value, as some authors have reported an association between preoperative symptoms and treatment success [12].

Only one study by Lipski et al. directly compared both restorative strategies within the same study population [21]. That study found no statistically significant difference in success rates between delayed restoration using Biodentine as a temporary bulk material and immediate definitive composite restoration [21]. In all other cases, comparisons were based on separate studies with different patient characteristics, treatment protocols, follow-up periods, and outcome definitions [1,11,12,13,14,15,16,17,18,19,20,22,23]. Therefore, the reported success rates should not be interpreted as evidence that one restorative approach is superior to the other. The highest success rates were reported in studies with moderate and high risk of bias, whereas the lowest success rates were observed across all risk levels, including studies with low, moderate, and high risk of bias.

A high level of heterogeneity was observed across the included studies. Differences were found in patient age, tooth type, stage of root development, inclusion criteria, caries removal techniques, disinfection and bleeding control methods, timing of restoration, and follow-up duration [1,11,12,13,14,15,16,17,18,19,20,21,22,23]. Outcome assessment also varied between studies. While some studies relied mainly on clinical findings and pulp sensibility tests [16], others additionally included radiographic evaluation [1,11,12,13,14,15,17,18,19,20,21,22,23]. These differences make direct comparison difficult and may partly explain the variation in reported success rates. The location of pulp exposure may also be clinically relevant, since occlusal exposures may provide more favorable isolation and sealing conditions than proximal or cervical exposures [21]. However, this factor was not consistently reported across the included studies.

Clinical procedural variables may also have influenced treatment outcomes. Across the included studies, higher success rates appeared to be most consistently associated with favorable baseline and procedural conditions, including a diagnosis of normal pulp or reversible pulpitis, absence of spontaneous pain or periapical pathology, small pulp exposures, adequate rubber-dam isolation, and the ability to achieve hemostasis within a limited time. However, because these variables were not uniformly reported or directly compared across studies, they should be regarded as probable clinical indicators of favorable prognosis rather than confirmed independent predictors of success. Caries removal strategies remain debated. Some authors recommend more complete removal of infected and affected dentine to reduce bacterial load [26,27], whereas current ESE guidance suggests that complete excavation in deep lesions may lead to unnecessary pulp exposure and should be avoided [24]. In the present review, both selective and non-selective approaches were represented, but the excavation methods were often insufficiently described. As a result, the influence of caries removal strategy on clinical success could not be reliably assessed. Similarly, the size of pulp exposure and the ability to achieve bleeding control were not consistently reported, despite their likely relevance to pulpal healing. Smaller exposures are generally associated with more localized inflammation and better healing potential [14], while the time required to obtain hemostasis has been suggested as an indirect indicator of pulp status.

Currently, there is very limited information in the literature regarding prognostic factors for indirect pulp capping. In a study by Alovisi et al., the risk of pulp necrosis was found to be statistically significantly higher in patients older than 40 years, as well as when self-etch adhesive systems were used [28]. According to Frankenberger et al., the size of the carious cavity may also have a statistically significant influence on the success of indirect pulp capping [29]. The success of direct pulp capping (DPC) depends on several clinical and procedural factors. The most important determinants include accurate pulp diagnosis, effective control of hemostasis, use of appropriate capping materials such as MTA or Biodentine, and the quality of the final coronal restoration [30]. Smaller exposure size and proper isolation may further improve outcomes, although their influence is less consistent. In contrast, patient age and root maturation appear to be less reliable predictors of treatment success [30]. However, age may still be considered a prognostic factor for DPC success. In a study by Lipski et al., the success rate of direct pulp capping in patients younger than 40 years reached 90.9% [21]. Prasertsuksom et al. found that the use of a rubber dam during direct pulp capping is associated with higher treatment success [31]. Cho et al. reported that age (<40 years), occlusal pulp exposure, and the use of bioceramic capping material had a statistically significant impact on pulp survival following direct pulp capping [32].

The timing of definitive restoration may be an important clinical factor. In delayed protocols, Biodentine is used as a temporary bulk restoration before placement of the final composite. This approach may allow additional maturation of the material and provide an opportunity to reassess pulpal status before definitive restoration. However, it may also increase the risk of restoration failure, patient noncompliance, and bacterial contamination if the temporary seal is compromised. In contrast, immediate composite restorations provide a definitive seal in a single visit and may reduce the risk of coronal microleakage associated with delayed treatment. Despite these theoretical differences, the available clinical evidence does not demonstrate a clear advantage of either strategy.

Differences in restorative techniques were also observed, including the use of an intermediate glass ionomer cement layer before composite placement in some studies [21,23]. Although glass ionomer cement may affect moisture control and marginal adaptation, its use was inconsistent across the included studies, and no clear effect on clinical outcomes could be identified. Experimental studies have suggested possible interactions between glass ionomer cement and calcium silicate-based materials during the early setting phase [33], but these effects were not directly evaluated in the included clinical studies. Similarly, polymerization heat, polymerization shrinkage, and the maturation-dependent bond strength of Biodentine may theoretically influence the quality of the coronal seal or the stability of the restoration, there is currently insufficient clinical evidence to confirm that these factors directly affect clinical outcomes.

Although both restorative approaches showed generally high success rates, failures were still reported. Possible reasons include inaccurate initial assessment of pulp status, inadequate coronal sealing, and patient-related factors [13,21,22]. However, because of inconsistent reporting and study variability, it was not possible to identify reliable predictors of failure. Interpretation of success was further complicated by the fact that pulp sensibility testing was commonly used as a primary outcome measure. Such tests may not always accurately reflect the true biological condition of the pulp, and false-negative results may occur, particularly during follow-up after vital pulp therapy [34]. In addition, relatively short follow-up periods in several studies may have underestimated the true frequency of late failures. Longer observation periods may reveal additional treatment failures that are not detectable within 6 or 12 months.

Several limitations of the available evidence should therefore be acknowledged. Most included studies evaluated only one restorative strategy, resulting primarily in indirect comparisons. There was also marked heterogeneity in study design, patient characteristics, clinical protocols, and outcome definitions, which precluded meta-analysis. The certainty of evidence was mainly downgraded because most included studies were judged to have a moderate risk of bias and the comparison between restorative strategies was largely indirect, with only one study directly comparing both approaches within the same population. Inconsistency was considered less critical, as both immediate and delayed restorative protocols generally showed favorable outcomes across studies, however, imprecision remained a concern due to small sample sizes, variable follow-up periods, and the lack of pooled quantitative analysis.

The certainty of the evidence was low due to risk of bias, inconsistency, indirectness, and imprecision. Consequently, the current evidence is insufficient to determine whether immediate definitive composite restoration or delayed composite placement after temporary Biodentine bulk restoration provides superior clinical outcomes after Biodentine pulp capping in permanent teeth. In current clinical practice, the choice between immediate and delayed composite restoration should be individualized. Immediate restoration may be preferred when good isolation is achievable, the procedure can be completed predictably in one visit, or when a second appointment may be uncertain. A delayed approach may be useful when additional Biodentine maturation, symptom reassessment, or staged management is preferred. In practice, the strategy may also be influenced by time limitations, patient tolerance, limited ability to keep the mouth open for prolonged periods, reduced cooperation, mental or physical disability, or treatment of children. However, delayed protocols require a reliable temporary seal, patient compliance, and careful Biodentine reduction. Therefore, based on current low-certainty evidence, the decision should be guided by clinical and patient-related factors rather than by proven superiority of either strategy. Well-designed prospective clinical studies with standardized protocols and direct head-to-head comparisons are needed to clarify the optimal restorative approach.

## 5. Conclusions

The available low-certainty clinical evidence suggests that both immediate definitive composite restoration and delayed composite placement after temporary Biodentine bulk restoration may be associated with favorable clinical outcomes following Biodentine pulp capping. However, the evidence is limited by substantial heterogeneity and relies largely on indirect comparisons across distinct study populations. Therefore, current evidence does not support a firm conclusion regarding the superiority of either restorative strategy. This conclusion remained unchanged when considering only low- or moderate-risk studies. Well-designed prospective head-to-head comparative studies with standardized protocols and longer follow-up are needed.

## Figures and Tables

**Table 1:** Search strategies in databases and the number of results found.

Data Base	Search Query	Result
PubMed	#1 (“pulp capping” OR “direct pulp capping” OR “indirect pulp capping” OR “vital pulp therapy” OR “reversible pulpitis treatment” OR “pulp exposure”) #2 (“Biodentine” OR “calcium silicate cement” OR “tricalcium silicate” OR “bioceramic cement”) #3 (“coronal restoration” OR “permanent restoration” OR “definitive restoration” OR “composite restoration” OR “composite” OR “temporary restoration” OR “temporary filling”) #4 (“success rate” OR “pulp sensibility” OR “clinical outcome” OR “radiographic outcome” OR “dentin bridge formation”) #5 #1 AND #2 AND #3 AND #4	20
Web of Science	(“pulp exposure”) AND (“pulp capping” OR “vital pulp therapy”) AND (“Biodentine” OR “composite”) AND (“outcome”)	102
Scopus	(“pulp exposure”) AND (“pulp capping” OR “vital pulp therapy”) AND (“Biodentine” OR “composite”) AND (“outcome”)	53
ScienceDirect	(“pulp exposure” OR “pulp capping” OR “vital pulp therapy”) AND (“Biodentine” OR “tricalcium silicate cement”) AND (“composite” OR “composite restoration” OR “temporary restoration”) AND (“success rate” OR “pulp sensibility” OR “clinical outcome” OR “radiographic outcome”)	379
Google Scholar	(“pulp exposure” OR “pulp capping” OR “vital pulp therapy”) AND (“Biodentine” OR “tricalcium silicate cement”) AND (“composite” OR “composite restoration” OR “temporary restoration”) AND (“success rate” OR “pulp sensibility” OR “clinical outcome” OR “radiographic outcome”)	504
Cochrane Library	#1 (“pulp capping” OR “vital pulp therapy” OR “pulp exposure”) #2 (“Biodentine” OR “calcium silicate cement” OR “tricalcium silicate”) #3 (“composite restoration” OR composite OR “temporary restoration” OR restoration #4 (“success rate” OR “pulp vitality” OR “clinical outcome” OR “radiographic outcome” OR “dentin bridge formation”) #5 #1 AND #2 AND #3 AND #4	78

**Table 2:** Inclusion and exclusion criteria.

Inclusion Criteria	Exclusion Criteria
Clinical studies evaluating indirect pulp capping (IPC) or direct pulp capping (DPC) using Biodentine *	Studies involving pulp exposure due to trauma or non-caries-related causes
Studies involving mature or immature permanent teeth	Non-human studies
Studies involving deep carious lesions in permanent teeth treated with indirect and/or direct pulp capping using Biodentine	Case reports, reviews, and meta-analyses
Studies evaluating clinical and/or radiographic outcomes	
Studies with a minimum follow-up period of 6 months	
Studies where permanent restoration was performed using composite resin or temporary Biodentine restoration	

* Studies reporting combined results for IPC and DPC were included only if data for each procedure could be clearly distinguished and extracted separately. If separate data were not available, such studies were excluded.

**Table 3:** Methodological characteristics of the included studies.

Study, Country	Study Design	Pulp Capping	Teeth Included	Caries Removal	Hemostasis (Solution; Duration)	Definitive Restoration Type and Timing	Follow-Up
Drouri et al. (2023, Morocco) [1]	Cohort	IPC DPC	43 teeth; mature; anterior, premolar, and molar teeth	Not specified	2.5% NaOCl + saline; Not specified	Biodentine bulk → composite (2 weeks)	1, 3, 6 months
Zanini et al. (2025, France) [16]	Cohort	DPC	36 teeth; mature; anterior and posterior teeth	Complete or partial	2% CHX; ≤5 min	Immediate composite	1, 2, 3 years
Hashem et al. (2015, UK) [12]	RCT	IPC	36 teeth; mature; anterior and posterior teeth	Selective (slow-speed rose-head burs + Carisolv)	Not specified	Biodentine bulk → composite (1 month)	1, 6, 12 months
Hashem et al. (2019, UK) [11]	RCT	IPC	36 teeth; mature; anterior and posterior teeth	Selective (slow-speed rose-head burs + Carisolv)	Not specified	Biodentine bulk → composite (1 month)	1, 6, 12, 24 months
Yavuz et al. (2025, Turkey) [20]	RCT	IPC DPC	48 teeth; mature; posterior teeth	Not specified	Not specified; ~5 min	Immediate composite	6 & 12 months
Parinyaprom et al. (2018, Thailand) [19]	RCT	DPC	29 teeth; immature and mature; tooth type not specified	Not specified	2.5% NaOCl; ≤10 min	Immediate composite	every 6 months Up to ~24 months
Awawdeh et al. (2018, Jordan) [22]	RCT	DPC/partial pulpotomy if bleeding persisted	34 teeth; mature; anterior and posterior teeth	Not specified	5% NaOCl; 3 min	Biodentine bulk → composite (1 week)	6, 12, 24, 36 months
Brizuela et al. (2017, Chile) [23]	RCT	DPC	60 teeth; immature and mature; permanent molars	Not specified	Saline; ≤10 min	Immediate composite (+GIC)	1 week, 3, 6, 12 months
Katge et al. (2017, India) [14]	RCT	DPC	29 teeth; immature; first permanent molars	Stepwise (rotary + spoon excavation)	Saline → 3% NaOCl; Not specified	Biodentine bulk → composite (3 months)	6 & 12 months
Peskersoy et al. (2021, Turkey) [18]	RCT	DPC	105 teeth; mature; permanent molars	Not specified	2.5% NaOCl → saline; 5 min	Immediate composite	1 month, 6 months, 1 & 3 years
Hegde et al. (2017, India) [17]	Cohort	DPC	12 teeth; mature; permanent molars	Not specified	3% NaOCl; Not specified	Biodentine bulk → composite (3 weeks)	3, 6, 12 months
Kusumvalli et al. (2019, India) [15]	Cohort	IPC DPC	12 teeth; mature; posterior teeth	Stepwise (rotary + spoon excavation)	Moist cotton pressure (unspecified) + 3% NaOCl; 1–2 min	Immediate composite	1, 3, 6, 12 months
Linu et al. (2017, India) [13]	Cohort	DPC	15 teeth; mature; mandibular permanent molars	Not specified	5% NaOCl; 10 min	Biodentine bulk → composite (2 weeks)	1, 3, 6, 12, 18 months
Lipski et al. (2018, Poland) [21]	Cohort	DPC	112 teeth; immature and mature; anterior and posterior teeth	Not specified	Saline/2% NaOCl/2% CHX; Not specified	Immediate composite (+GIC) or delayed composite (2–3 months)	2–3 months & 1–1.5 years

**Table 4:** JBI critical appraisal tool for Cohort studies.

Study	Lipski, 2018 [21]	Linu, 2017 [13]	Kusumvalli, 2019 [15]	Hegde, 2017 [17]	Zanini, 2025 [16]	Drouri, 2023 [1]
1. Were the two groups similar and recruited from the same population?	−	+	−	+	+	+
2. Were the exposures measured similarly to assign people to both exposed and unexposed groups?	o	+	o	+	+	+
3. Was the exposure measured in a valid and reliable way?	+	+	+	+	+	+
4. Were confounding factors identified?	o	+	−	o	o	o
5. Were strategies to deal with confounding factors stated?	−	−	−	−	−	−
6. Were the groups/participants free of the outcome at the start of the study (or at the moment of exposure)?	+	+	+	+	+	+
7. Were the outcomes measured in a valid and reliable way?	+	+	+	+	+	o
8. Was the follow up time reported and sufficient to be long enough for outcomes to occur?	+	+	+	+	+	o
9. Was follow up complete, and if not, were the reasons to loss to follow up described and explored?	+	o	o	+	−	+
10. Were strategies to address incomplete follow up utilized?	o	−	−	o	+	−
11. Was appropriate statistical analysis used?	+	+	+	+	+	o
Risk of bias	Moderate	Moderate	Moderate	Moderate	Moderate	Moderate

+ yes; − no, o—non applicable/not clear.

**Table 5:** JBI critical appraisal tool for RCT.

Study	Hashem, 2015 [12]	Hashem, 2019 [11]	Brizuela, 2017 [23]	Awawdeh, 2018 [22]	Katge, 2017 [14]	Parinyaprom, 2018 [19]	Yavuz, 2025 [20]	Peskersoy, 2021 [18]
1. Was true randomization used for assignment of participants to treatment groups?	+	+	+	+	−	+	+	+
2. Was allocation to treatment groups concealed?	+	+	o	−	−	o	o	o
3. Were treatment groups similar at the baseline?	+	+	+	o	+	+	+	o
4. Were participants blind to treatment assignment?	o	o	o	+	o	+	o	o
5. Were those delivering treatment blind to treatment assignment?	−	−	−	−	−	−	−	−
6. Were outcomes assessors blind to treatment assignment?	+	+	o	+	o	+	+	o
7. Were treatment groups treated identically other than the intervention of interest?	+	+	+	+	o	+	+	+
8. Was follow up complete and if not, were differences between groups in terms of their follow up adequately described and analyzed?	o	+	−	+	−	+	+	+
9. Were participants analyzed in the groups to which they were randomized?	+	+	+	o	+	+	+	+
10. Were outcomes measured in the same way for treatment groups?	+	+	+	+	+	+	+	+
11. Were outcomes measured in a reliable way?	+	+	+	+	o	+	o	o
12. Was appropriate statistical analysis used?	+	+	+	+	+	+	+	+
13. Was the trial design appropriate, and any deviations from the standard RCT design (individual randomization, parallel groups) accounted for in the conduct and analysis of the trial?	+	+	+	+	o	+	+	+
Risk of bias	Low	Low	Moderate	Moderate	High	Low	Moderate	High

+ yes; − no, o—non applicable/not clear.

**Table 6:** GRADE summary of findings.

Restorative Strategy	Number of Studies	Number of Cohort Studies	Number of RCT Studies	Success Range (%)	Consistency of the Findings Across the Studies	Main Limitations	Overall Certainty of Evidence
IPC immediate	2	1	1	80–95.2%	Consistent	Very small number of studies; small sample sizes; indirect comparison only; short follow-up; heterogeneity in clinical protocols	Low
IPC delayed	3	1	2	77.8–88%	Consistent	Limited number of studies; some follow-up losses or incompletely separated data; indirect comparison only; variation in follow-up duration and outcome criteria	Low
DPC immediate	7	3	4	79–100%	Consistent	Heterogeneity in patient age, tooth type, pulp exposure size, hemostasis protocol, follow-up duration, and outcome assessment; mostly indirect comparisons; some studies with moderate/high risk of bias	Low
DPC delayed	6	4	2	74–100%	Consistent	Heterogeneity in clinical protocols and timing of final restoration; mostly indirect comparisons; variable follow-up; some incomplete or non-separately reported follow-up losses; some studies with moderate/high risk of bias	Low

**Table 7:** Clinical outcomes of direct pulp capping using Biodentine as a bulk restoration.

Study	Sample Size	Follow-Up	Success (%)	Evaluation Criteria	Failures	Follow-Up Losses
Drouri et al. (2023) [1]	23 teeth	6 months	74%	Asymptomatic; positive pulp sensibility test; no radiographic pathology	6 teeth	Not separately reported
Lipski et al. (2018) [21]	37 teeth	1–1.5 years	78.4%	Asymptomatic; positive pulp sensibility test; no radiographic pathology	8 teeth	Not separately reported
Awawdeh et al. (2018) [22]	34 teeth	36 months	91.7%	Asymptomatic; positive pulp sensibility test; no swelling/fistula; no radiographic pathology	5 teeth	8 teeth
Linu et al. (2017) [13]	15 teeth	18 months	92.3%	Asymptomatic; positive pulp sensibility test; dentin bridge formation; no radiographic pathology	1 tooth	2 teeth
Katge et al. (2017) [14]	29 teeth	12 months	100%	Asymptomatic; positive pulp sensibility test; dentin bridge; no periapical pathology	Not reported	8 teeth
Hegde et al. (2017) [17]	12 teeth	12 months	83.3%	Asymptomatic; no percussion sensitivity; no swelling/fistula; no radiographic pathology	2 teeth	Not reported

**Table 8:** Clinical outcomes of direct pulp capping followed by immediate permanent composite restoration.

Study	Sample Size	Follow-Up	Success (%)	Evaluation Criteria	Failures (n)	Follow-Up Losses
Zanini et al. 2025 [16]	36 teeth	3 years	86.0%	Asymptomatic; positive pulp sensibility test; no periapical pathology; acceptable restoration margins.	8 teeth	10 teeth
Lipski et al. 2018 [21]	49 teeth	1–1.5 years	85.7%	Asymptomatic; positive pulp sensibility test; no radiographic pathology	7 teeth	Not reported
Parinyaprom et al. 2018 [19]	29 teeth	18.9 ± 12.9 months	96.4%	Asymptomatic; positive pulp sensibility test; no periapical pathology	Not reported	1 tooth
Brizuela et al. 2017 [23]	60 teeth	12 months	100%	Asymptomatic; positive pulp sensibility test; no radiographic pathology	Not reported	35 teeth
Yavuz et al. 2025 [20]	26 teeth	12 months	96%	Asymptomatic; no percussion pain; no radiographic pathology	2 teeth	2 teeth
Kusumvalli et al. 2019 [15]	7 teeth	12 months	85.7%	Asymptomatic; positive pulp sensibility test; no radiographic pathology	1 tooth	Not reported
Peskersoy et al. 2021 [18]	105 teeth	3 years	79%	Asymptomatic; positive pulp sensibility test; no radiographic pathology	21 teeth	Not separately reported

**Table 9:** Clinical outcomes of indirect pulp capping using Biodentine as a bulk restoration.

Study	Sample Size	Follow-Up	Success (%)	Evaluation Criteria	Failures (n)	Follow-Up Losses
Drouri et al. 2023 [1]	17 teeth	6 months	88%	Asymptomatic; positive pulp sensibility test; no radiographic pathology	2 teeth	Not reported
Hashem et al. 2019 [11]	36 teeth	24 months	77.8%	Asymptomatic; positive pulp sensibility test; no percussion sensitivity; no radiographic pathology	6 teeth	9 teeth
Hashem et al. 2015 [12]	36 teeth	12 months	83.3%	Asymptomatic; positive pulp sensibility test; no swelling/fistula; no radiographic pathology	Not reported	Not reported

**Table 10:** Clinical outcomes of indirect pulp capping followed by immediate permanent composite restoration.

Study	Sample Size	Follow-Up	Success (%)	Evaluation Criteria	Failures (n)	Follow-Up Losses
Kusumvalli et al. 2019 [15]	5 teeth	12 months	80%	Asymptomatic; positive pulp sensibility test; no radiographic pathology	1 tooth	Not reported
Yavuz et al. 2025 [20]	22 teeth	12 months	95.2%	Asymptomatic; no percussion pain; no radiographic pathology	2 teeth	2 teeth

## Data Availability

No new data were created in this study. Data sharing is not applicable to this article.

## Supplementary Materials

The following supporting information can be downloaded at: https://www.mdpi.com/article/10.3390/jfb17050241/s1, File S1: PRISMA checklist.
